# Comparison of RBE values of high- LET α-particles for the induction of DNA-DSBs, chromosome aberrations and cell reproductive death

**DOI:** 10.1186/1748-717X-6-64

**Published:** 2011-06-08

**Authors:** Nicolaas AP Franken, Rosemarie ten Cate, Przemek M Krawczyk, Jan Stap, Jaap Haveman, Jacob Aten, Gerrit W Barendsen

**Affiliations:** 1 Department of Radiation Oncology, Laboratory for Experimental Oncology and Radiobiology (LEXOR), Centre for Experimental Molecular Medicine, University of Amsterdam, PO Box 22700, 1100 DE Amsterdam, The Netherlands; 2 Department of Cell Biology and Histology, Academic Medical Centre, University of Amsterdam, PO Box 22700, 1100 DE Amsterdam, The Netherlands

## Abstract

**Background:**

Various types of radiation effects in mammalian cells have been studied with the aim to predict the radiosensitivity of tumours and normal tissues, e.g. DNA double strand breaks (DSB), chromosome aberrations and cell reproductive inactivation. However, variation in correlations with clinical results has reduced general application. An additional type of information is required for the increasing application of high-LET radiation in cancer therapy: the Relative Biological Effectiveness (RBE) for effects in tumours and normal tissues. Relevant information on RBE values might be derived from studies on cells in culture.

**Methods:**

To evaluate relationships between DNA-DSB, chromosome aberrations and the clinically most relevant effect of cell reproductive death, for ionizing radiations of different LET, dose-effect relationships were determined for the induction of these effects in cultured SW-1573 cells irradiated with gamma-rays from a Cs-137 source or with α-particles from an Am-241 source. RBE values were derived for these effects. Ionizing radiation induced foci (IRIF) of DNA repair related proteins, indicative of DSB, were assessed by counting gamma-H2AX foci. Chromosome aberration frequencies were determined by scoring fragments and translocations using premature chromosome condensation. Cell survival was measured by colony formation assay. Analysis of dose-effect relations was based on the linear-quadratic model.

**Results:**

Our results show that, although both investigated radiation types induce similar numbers of IRIF per absorbed dose, only a small fraction of the DSB induced by the low-LET gamma-rays result in chromosome rearrangements and cell reproductive death, while this fraction is considerably enhanced for the high-LET alpha-radiation. Calculated RBE values derived for the linear components of dose-effect relations for gamma-H2AX foci, cell reproductive death, chromosome fragments and colour junctions are 1.0 ± 0.3, 14.7 ± 5.1, 15.3 ± 5.9 and 13.3 ± 6.0 respectively.

**Conclusions:**

These results indicate that RBE values for IRIF (DNA-DSB) induction provide little valid information on other biologically-relevant end points in cells exposed to high-LET radiations. Furthermore, the RBE values for the induction of the two types of chromosome aberrations are similar to those established for cell reproductive death. This suggests that assays of these aberrations might yield relevant information on the biological effectiveness in high-LET radiotherapy.

## Background

The individualization of cancer treatment by fractionated application of ionizing radiation is expected to benefit from a rapid assessment of the radiosensitivity of clonogenic cells in a biopsy obtained before the treatment starts, or of the effectiveness of the first fraction dose of a schedule for damage to cells in a biopsy obtained after this fraction [1].

The measurement of clonogenic capacity of the cells, although it is the most relevant endpoint, requires several weeks of culturing and is likely to depend on selection of cells in adapting to culture media.

The measurement of chromosome aberrations (CA) in mitotic cells as a marker of radiosensitivity may be subject to selection because damaged cells may not all proceed equally rapidly to mitosis. However, the technique of premature chromosome condensation might provide an applicable alternative, because the analysis can be performed rapidly without the requirement of cells entering into mitosis [2-5].

Another recently developed rapid technique of assessment of cell damage that has been suggested to provide information on radiosensitivity involves the measurement of ionising radiation induced foci (IRIF) of DNA repair-related proteins accumulating at DNA double-strand breaks (DSB) [6]. However, the quantitative relationship between the IRIF induction and biologically relevant endpoints is not yet clear [7-10].

In the application of high-LET radiations to the treatment of cancer an additional type of quantitative information is required: the relative biological effectiveness value (RBE). This is especially relevant with the increasing application of external radiotherapy with light ion beams and of alpha-particle emitters in targeted radionuclide therapy [11-15],

The mechanisms by which ionizing radiations produce chromosome aberrations and reproductive death in mammalian cells are insufficiently elucidated to derive quantitative information applicable to the design of individualized cancer treatments, because this requires data about relevant α and β values and their ratio in the biophysical linear-quadratic model. These parameters are differently influenced by repair mechanisms in various cell types and by the linear energy transfer of the radiation (LET) [16-20].

Various mechanisms of damage induction by ionising radiations have been proposed that might explain the high RBE values of high-LET radiations.

Among the many types of DNA damage that are induced by ionising radiation in mammalian cells, DSB are generally recognized as the major initial lesions that can result in chromosome aberrations and impairment of the reproductive integrity which are relevant in clinical radiotherapy. However, a simple direct causal relationship between these effects cannot be inferred, because the number of DNA-DSB produced by a dose of 1 Gy of low LET ionizing radiation is much larger than the numbers of induced chromosome aberrations or cell reproductive death [21]. A large majority of the induced DSB is known to be repaired by non-homologous end joining (NHEJ) or homologous recombination (HR), but characteristics of the minority of DSB which yield biological damage are still subject of studies [22]. Furthermore the strong dependence of frequencies of chromosome aberrations and cell inactivation on the linear energy transfer (LET) of ionizing particles, with maximum values of RBE in the range from 5-20 compared to γ-rays, is not observed for DNA-DSBs [16-19]. In a review by Prise *et al *the authors conclude that the RBE values of high LET radiations, i.e. LET's in the range of 50-200 keV/μm, are between 1-2 for DSBs, although at low doses of 1-5 Gy data were not considered sufficiently accurate [23]. New methods of DNA-DSB detection also applied in our studies, can provide more accurate data in the range of low doses of 1 to 5 Gy [8,9,24,25].

Two possible explanations for the discrepancy between the RBE for DSB induction by high-LET radiations compared to other biologically-relevant end points have been advanced.

First, high-LET radiations might cause more complex damage in DNA, which might be less easily repaired by mechanisms in cells [26]. However, it was also suggested that the number rather than the molecular structure of DSB is more important for the formation of chromosomal aberrations [27]. The second explanation for the high RBE of high-LET radiations is that interaction of two or more DSB produced in close proximity results in a high probability for induction of chromosome aberrations and cell reproductive inactivation. High-LET particles produce many DSB along their tracks in cells within distances of less than 1-2 micrometers and this might result in an increased probability for interaction and enhanced formation of chromosome aberrations that are known to correlate with cell reproductive death [16-19]. Support for this explanation was provided by the observation of clustering of chromosome domains containing DSB induced by high-LET alpha particles and visualised as IRIF [28,29]. Due to the localization of the DSB along the straight tracks of the particles the probability of clustering and interaction is higher than for low-LET radiations. It is important to note that not all chromosome aberrations cause cell reproductive death and therefore the RBE values for these two effects might be different. If all chromosome breaks detected by the premature chromosome condensation technique (PCC) were to cause cell reproductive death, mammalian cells would be more sensitive to inactivation by at least a factor 5 [21]. Thus lethal aberrations might be induced with a higher or lower RBE by high-LET radiations than all aberrations but little information on these differences has been reported. In a recent review the observation is made that RBE values ranging between 2 and 30 have been reported in studies applying premature chromosome condensation (PCC) and fluorescence in situ hybridization (FISH) [30].

Because the differences in RBE values for various effects of high-LET radiations in cells cannot be derived quantitatively on the basis of the known mechanisms, relevant data can be obtained from cell line based experiments. This is the purpose of experiments reported in the present communication.

## Materials and methods

### Cell culture

The human squamous cell lung carcinoma derived line SW-1573 was grown at 37°C as monolayer in 75 cm^2 ^tissue culture flasks (Costar) in Leibowitz-15 medium (L-15, Gibco-brl Life Technologies, Breda, The Netherlands) supplemented with 10% heat inactivated fetal bovine serum and 2 mM glutamine, 100 U/ml penicillin and 100 μg/ml streptomycin (Gibco), in an atmosphere of 0% CO_2. _The doubling time of the cells during exponential growth is 22-24 hour [31-33]. Cells were irradiated in plateau phase and immediately used either for DNA-DSB detection, clonogenic assay or premature chromosome condensation metaphase preparation. Cell cycle distribution was monitored by flow cytometry and at the time of irradiation over 90% of cells was in G_0_+G_1 _phase. For irradiation with γ-rays cells are grown in 6 cm diameter culture dishes.

For the α particle irradiation cells were cultured in custom made dishes with 2 μm thick mylar bottoms and 6 cm diameter [34,35].

### Irradiation

Plateau phase cell cultures were exposed to α radiation from an ^241^Am source or γ-rays from a ^137^Cs source.

The 11 MBq ^241^Am-source was located at a distance of 50 mm underneath the dishes and the particles passed through 50 mm of helium and the thin mylar bottom of the culture dishes before entering the cells through the bottom of the dishes with about 4 MeV energy and a residual range of 25 μm in tissue. The mean LET of particles reaching the cells is 130 keV/μm [35]. The dose rate was measured with a custom made ionization chamber as described in earlier publications [35]. The length of α particle paths in cell nuclei was about 5 μm. The average dose rate in the cell nucleus was 0.20 Gy/min. Doses of up to 1.6 Gy were used for determining survival, 1.4 Gy for γ-H2AX foci numbers and of up to 0.8 Gy for determining chromosomal aberrations.

The dose rate of the ^137^Cs source used was 0.6 Gy/min. For induction of γ-H2AX foci, chromosome aberrations and cell reproductive death doses of up to respectively 1.4, 4.0 and 8.0 Gy were used.

### Clonogenic assay

Directly after irradiation cells were trypsinized and replated for clonogenic survival assay in appropriate cell numbers in 6-well macroplates [36]. Subsequently, cells were incubated for 10 days. Surviving colonies were fixated and stained with glutaraldehyde-crystal violet solution and counted. Survival curves were analyzed using SPSS (Chicago, IL, USA) statistical software by means of fit of data by weighted linear regression, according to the linear-quadratic formula: S(D)/S(0) = exp-(αD+βD^2^) [20,36,37].

### Chromosomal aberrations

Chromosomal aberrations were studied in prematurely condensed chromosomes (PCCs). For induction of PCCs, 80 nM of Calyculin A was added for 1 h immediately after irradiation [2,5,38]. Visualization of chromosomes was accomplished by fluorescent in situ hybridization (FISH). Cells were harvested, treated with hypotonic KCl solution (0.075 M) for 20 min and fixed in methanol/acetic acid (3:1). Finally the cell suspension was dropped on precleaned slides and air-dried. PCC spreads were hybridized to whole chromosome-specific FITC labeled probes for chromosome 2 (Metasystems) using the method described earlier [4,38,39]. Slides were counterstained with DAPI (2.5 μg/ml) and embedded in antifade solution (Vecta shield, Vector laboratories, Burlingame, CA. USA).

The SW-1573 cells contain between 60 and 67 chromosomes. To study the relationship between the yield of exchanges and radiation doses, chromosome 2 was selected. This chromosome exhibits no spontaneous exchanges and three copies of chromosome 2 were present in over 95% of the metaphases studied. According to the chromosome length measurements the relative lengths of chromosomes 2 are 7.8 ± 0.6% of the complete genome [40]. Slides were examined using a fluorescence microscope (Axioskop 2 MOT Zeiss, Jena, Germany) equipped with suitable filter block to detect the painted chromosomes (FITC and DAPI for total DNA) in one image. Two to four hundred PCCs were scored for each dose. The induction of colour junctions and chromosome fragments of painted chromosomes was scored according to the method described by Tucker *et al *[41]. An exchange between a fragment of a painted chromosome and a fragment of an unpainted chromosome was scored as a colour junction. Rejoining of two identically painted chromosome fragments without a centromere was scored as fragment.

### Detection of γ-H2AX: Immunohistochemistry and scoring

To detect γ-H2AX foci which are formed at sites of DSB, cells were grown on plastic cover slips. The cover slips (22 × 26 mm) were sterilized with alcohol (70%) and were placed in 60 mm cell culture dishes. The cells were reseeded at a density of 2.5 × 10^5 ^cells in cell culture dishes containing sterile cover slips and were grown until a confluent layer was obtained. The cells were then irradiated. For α-particle irradiation the cover slips were placed on a dish with a mylar bottom with the cells facing the mylar.

After α particle irradiation cells were fixated 5 min after treatment. After γ- irradiation cells were fixated 30 min after treatment. At these time intervals maximal number of foci were counted. After irradiation, cells were washed with PBS and fixated in PBS containing 2% paraformaldehyde for 15 min. After three further washes in PBS cells were treated with PBS containing 0.1% Triton X-100 & 1% FCS (TNBS) for 30 min.

The primary antibody used was a mouse monoclonal anti-γ-H2AX (Millipore, Ca) diluted 1:100 in TNBS. Permeabilized cells were incubated with 50 μl primary antibody under a parafilm strip for 90 min. at room temperature. Cells were then washed with PBS for about 5 min and the parafilm strip was removed. After this, cells were washed 2 times with TNBS. The secondary antibody used was a Goat anti-Mouse Cy3 (Jackson, Immunoresearch, Europe Ltd, Suffolk, UK)) also diluted 1:100 in TNBS.

Cells on cover slips were incubated with 50 μl secondary antibody under a parafilm strip for 30 min. at room temperature. Cells were then washed 3 times with TNBS for about 5 min and the parafilm strip was removed at the first wash. Nuclei were stained with DAPI (2.5 μg/ml) and subsequently embedded in vectashield.

Digital image analysis was performed to determine the number of γ-H2AX IRIF (ionizing radiation induced foci). Fluorescent photomicrographs of γ-H2AX foci were obtained using Image Pro Plus software. Stack images of cells were obtained using a Leica DM RA HC Upright Microscope equipped with a CCD camera. Stack images of 100 cells per sample were taken using Image pro plus software. One stack image consists of 40 slices with a 200 nm interval between the slices along the z-axis. Images were then processed and the number of foci in cells was scored using custom made software [28,29].

All experiments were carried out in triplicates, independently from each other. Numbers of foci in unirradiated control cells were subtracted from numbers in irradiated samples.

S-phase cells were excluded as an EDU staining (Invitrogen, Eugene, Oregon USA) was used to mark these cells.

## Results

The PCC technique was applied to measure induction of chromosome aberrations, induction of DSB was estimated by scoring γ-H2AX IRIF and reproductive cell death was measured by clonogenic assay.

**Figure 1:**
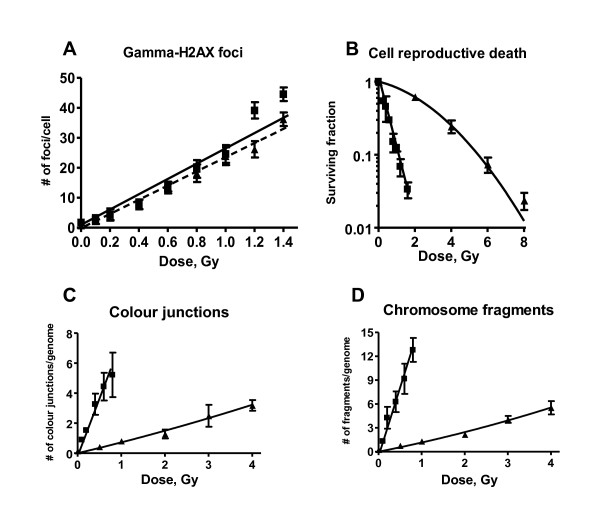
**Number of γ-H2AX foci (A), Radiation dose survival curves (B), frequency of colour junctions (C) and chromosome fragments (D) for SW1573 cells after α particle (black squares) and gamma irradiation (black triangles)**. Calculated RBE values for DNA-DSBs, cell reproductive death, chromosome fragments and colour junctions are 1.0 ± 0.3, 14.7 ± 5.1, 15.3 ± 5.9 and 13.3 ± 6.0 resp.

In figure 1 induction of Gamma-H2AX foci, radiation dose survival curves, frequencies of chromosome fragments and colour junctions in SW1573 cells after α- particle and γ-ray irradiation are presented. Figure 1A shows similar induction of DSB after α- particles and γ-rays at the time intervals studied. Figure 1B shows the radiation dose survival curves which demonstrate that cell reproductive death after α- particle radiation is much more frequently induced than after γ-ray irradiation. From figure 1C and 1D it can be observed that after α- particle radiation the induction of chromosomal fragments and colour junctions is much higher than after γ-ray irradiation. From these data the linear and quadratic parameters were derived by analysis with the formula S(D)/S(0) = exp-(αD+βD^2 ^) for cell survival curves and F(D) = αD+βD^2 ^for the induction of DSB, chromosome fragments end colour junctions. Except for cell survival curves for γ-irradiation, the values of the quadratic parameter, β, for DSB induction, chromosome fragments and colour junction formation were not significantly different from zero. Because with alpha radiation only linear parameters were derived, for assessment of RBE values the comparison with linear parameters for gamma radiation for all endpoints is appropriate. Therefore, in order to compare equivalent values only the values of α are considered for evaluation and discussion of RBE values. These values are summarized in table 1. The values for the quadratic parameters for cell survival, chromosomal fragments and colour junctions after γ-irradiation are 0.05 ± 0.01, 0.08 ± 0.08 and 0.03 ± 0.07 resp.

**Table 1:** Values of α of the LQ model for survival curves, chromosomal fragments, colour junctions and DNA DSBs of SW-1573 cells after alpha particle irradiation and after γ irradiation

	α-particle irradiation Gy^-1^	γ-irradiation Gy^-1^	RBE value
**Survival**	**2.2 ± 0.38**	**0.15 ± 0.045**	**14.7 ± 5.1**

**Chromosomal fragments**	**16.8 ± 4.5**	**1.1 ± 0.31**	**15.3 ± 5.9**

**Colour junctions**	**9.2 ± 3.2**	**0.69 ± 0.2**	**13.3 ± 6.0**

**DSB (Gamma-H2AX foci)**	**25 ± 8.2**	**25 ± 3.0**	**1.0 ± 0.3**

The chromosomal fragments, colour junctions were determined in chromosome 2 and the α-values are corrected for the DNA content of the complete genome. Survival curves were analyzed using S(D)/S(0) = exp-(αD+βD^2^) [20,36,37]. The values of α for chromosomal fragments colour junctions and DSB are calculated according to F(D) = αD+βD^2 ^[40]

## Discussion

For the survival curve, chromosomal fragments and colour junctions of cells irradiated with γ-rays linear-quadratic dose response curves were obtained while for DNA DSBs the dose-response effect relation is linear. After α- particle radiation the dose response curves for all endpoints studied were linear. Therefore, in order to derive relevant RBE values, only the parameters of the linear terms will be compared. To measure induction of chromosome aberrations, we applied premature chromosome condensation (PCC) because this method does not require the treated cells to proceed to mitosis, which may select for cells with less damage [2].

### Comparison of the frequencies of induced effects

The value of α for induction of DSB (table 1) is evidently much larger than the corresponding value for cell inactivation, leading to the conclusion that only a small fraction of the DSB (about 1% of DSB induced by γ-rays and about 10% by α-particles) are causing cell death. On the other hand, the values of α for formation of chromosome fragments and colour junctions as shown in table 1 are about 8 and 4 resp. times larger than the corresponding values for induction of cell reproductive death, for α- as well as for γ-radiation. This suggests that many of these aberrations are either repaired or do not cause complete impairment of the cell reproductive capacity. The number of fragments is higher than that of colour junctions as the induction of chromosomal aberrations was studied shortly after treatment and at that time point not all colour junctions might have been formed. It is generally observed that colonies arising from cells surviving irradiation are smaller, as compared to colonies formed by unirradiated cells, indicating that their genomes might be damaged, although their reproductive potential is not eliminated [42]. From analyses of cell survival curves derived for different particles in relation to LET, it has been earlier suggested that a contribution to the linear term is due to potentially lethal damage (PLD) [16]. The present results are compatible with this suggestion.

### Comparison of RBE values

The calculated RBE value of 1.0 ± 0.3 for induction of γ-H2AX foci is much smaller than the values for induction of cell reproductive death, chromosome fragments and colour junctions, which are not significantly different [43].

Although there is a clear correlation between cell reproductive death and the induction of chromosomal aberrations, a direct causal relationship between these effects cannot yet be inferred [44]. Further studies of RBE values at different time intervals post irradiation should yield information on this problem. The RBE value of 14.6 for cell reproductive death is similar to values in the range of 5 to 15 published for many other lines of cultured cells [45]. The RBE of 1 derived for the induction of DNA-DSB is consistent with published results obtained with other methods at higher doses as summarized by Prise *et al *[23]. However, data obtained by Prise *et al *using the filter elution technique show a significant contribution of the quadratic parameter β in the dose-effect curves at large doses of X-rays [44,45]. This observation is not incompatible with our data showing a linear dependence of the number of γ-H2AX foci on the dose of γ-rays at low doses. The α/β ratios that can be derived from the data obtained with the filter elution technique are equal to 27 Gy and 16 Gy for AL-K and 250 kV X-rays, respectively. From these large values it is evident that at doses in the range of up to 1.4 Gy as used in our studies the quadratic term contributes less then 10 percent to the total effect. This contribution is not detectable as a deviation from linearity in our results at low doses.

**Figure 2:**
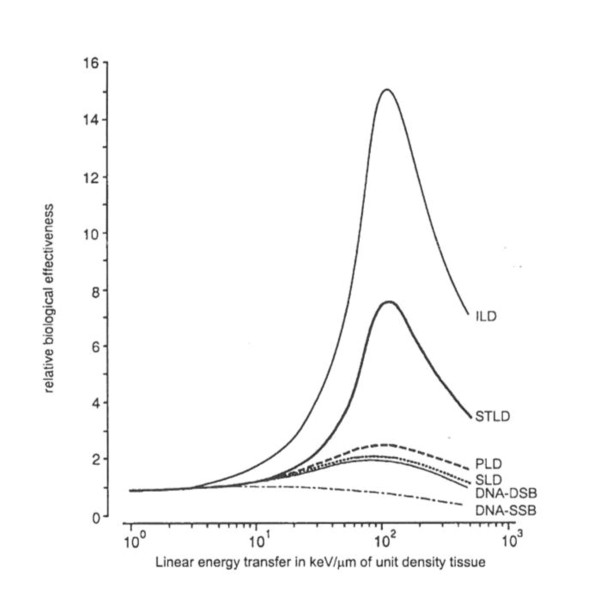
**Relative biological effectiveness (RBE) as a function of the linear energy transfer (LET) for different types of lethal damage in mammalian cells and for DNA damage**. ILD, irrepairable lethal damage, derived as the contribution to the linear parameter α of the LQ model that is not repaired after irradiation of cells, even if maintained in conditions optimal for repair. PLD, potentially lethal damage, derived as the contribution to the linear parameter α that after irradiation is repaired in conditions optimal for repair. STLD, single track lethal damage, derived as the linear parameter α in conditions in which PLD is not repaired. SLD, sublethal damage, derived from survival curves as the square root of the quadratic parameter β of the LQ model. DNA-DSB, RBE for double strand breaks in DNA., DNA-SSB, RBE for single strand breaks in DNA. (from Barendsen *et al*.) [46].

Based on the available literature, it can be suggested that the RBE for DSB induction increases as a function of LET between 20 and 80 keV/μm to about 2 and subsequently decreases to about 1 at larger LET (summarized in Figure 2) [16-18,46]. The curve presented in this figure for DNA-DSB induction was derived as an average of data published by different investigators, as summarised in reference 18. This figure is included to illustrate the small dependence of DNA-DSB on LET, but the absolute values may vary for different cell lines. The RBE value of 1 obtained for DSB induction by 130 keV/μm α-particles reported here is not inconsistent with these data.

## Conclusions

The final conclusion from the presented results is that assessment of the amount of DSB induced by ionizing radiation as measured by us shortly after radiation is unlikely to provide information about the biological effectiveness of high LET radiations of relevance in the treatment of cancer. This is in agreement with the report by Yoshikawa *et al *inferring that γ-H2AX IRIF numbers in tumour cells fail to correlate with their radiosensitivity [7]. On the other hand, the RBE values for induction of chromosome aberrations are quite similar to the value for cell reproductive death. This suggests that these end-points might be more appropriate in assessment of biological effectiveness of high-LET radiations. Recently it has been shown that PCC-FISH can be applied directly to biopsy cultures and biopsies derived from cervical cancer patients [2-4]. This technique might, therefore, yield relevant information on the effectiveness of high-LET radiations.

## Competing interests

The authors declare that they have no competing interests.

## Authors' contributions

NAPF performed the clonogenic survival assays, foci studies and coordinated the study. NAPF and GWB drafted the research, performed the dosimetry of the alpha particle irradiation and wrote the paper. RtC and JH performed the chromosomal aberration studies. PK, JS and JA helped with the discussion of the data. All authors read and approved the final manuscript.
